# Genomic and transcriptomic evidence of light-sensing, porphyrin biosynthesis, Calvin-Benson-Bassham cycle, and urea production in Bathyarchaeota

**DOI:** 10.1186/s40168-020-00820-1

**Published:** 2020-03-31

**Authors:** Jie Pan, Zhichao Zhou, Oded Béjà, Mingwei Cai, Yuchun Yang, Yang Liu, Ji-Dong Gu, Meng Li

**Affiliations:** 1 Shenzhen Key Laboratory of Marine Microbiome Engineering, Institute for Advanced Study, Shenzhen University, Shenzhen, China; 2 Laboratory of Environmental Microbiology and Toxicology, School of Biological Sciences, The University of Hong Kong, Pokfulam Road, Hong Kong, SAR China; 3 Faculty of Biology, Technion-Israel Institute of Technology, 32000 Haifa, Israel

**Keywords:** Bathyarchaeota, Rhodopsin, Porphyrin biosynthesis, Calvin-Benson-Bassham cycle, Urea producing, Trimethylamine degradation, Microoxic lifestyle

## Abstract

**Background:**

Bathyarchaeota, a newly proposed archaeal phylum, is considered as an important driver of the global carbon cycle. However, due to the great diversity of them, there is limited genomic information that accurately encompasses the metabolic potential of the entire archaeal phylum.

**Results:**

In the current study, nine metagenome-assembled genomes of Bathyarchaeota from four subgroups were constructed from mangrove sediments, and metatranscriptomes were obtained for evaluating their in situ transcriptional activities. Comparative analyses with reference genomes and the transcripts of functional genes posit an expanded role for Bathyarchaeota in phototrophy, autotrophy, and nitrogen and sulfur cycles, respectively. Notably, the presence of genes for rhodopsins, cobalamin biosynthesis, and the oxygen-dependent metabolic pathways in some Bathyarchaeota subgroup 6 genomes suggest a light-sensing and microoxic lifestyle within this subgroup.

**Conclusions:**

The results of this study expand our knowledge of metabolic abilities and diverse lifestyles of Bathyarchaeota, highlighting the crucial role of Bathyarchaeota in geochemical cycle.

Video abstract.

## Background

Bathyarchaeota, formerly named MCG (Miscellaneous Crenarchaeotal Group) [1], is a newly proposed archaeal phylum within the TACK (Proteoarchaeota) superphylum [2–4]. It is a cosmopolitan phylum, inhabiting various anoxic environments, such as groundwater, paddy soil, hot spring, salt marsh sediments, estuary, mangrove sediments, seafloor, and hydrothermal sediments [5–11]. It is also one of the most numerous archaeal groups in the marine sub-seafloor, estimated to have 2.0–3.9 × 10^28^ cells in the global ecosystem [3, 12]. The ubiquity and high abundance suggested that Bathyarchaeota might play a role in the global biogeochemical cycle [13]; however no pure cultures of Bathyarchaeota have been successfully established. Recently, an enrichment of Bathyarchaeota was obtained, suggesting the utilization of lignin as an energy source and bicarbonate as a carbon source by subgroup 8 (Bathy-8), yet more metabolisms need to be explored [14].

Based on the analysis of metagenome-assembled genomes (MAGs) and single-cell genomes (SAGs), Bathyarchaeota has been implicated to have potential abilities for CO_2_ fixation with Wood-Ljungdahl pathway, acetogenesis, methane metabolism, and degradation of peptides, fatty acids, aromatic, and other organic compounds [2, 3, 14–17], suggesting Bathyarchaeota may play an important role in the global carbon cycle. At least 25 subgroups have been identified in Bathyarchaeota based on the phylogenetic analyses of 16S rRNA genes [13], and many subgroups display distinct environmental preferences implicating diversification and adaptation to unique environmental conditions [6, 18–21]. Thus, the current information is too limited to comprehensively understand the metabolic capacities of Bathyarchaeota and its role in the geochemical cycle.

Bathyarchaeota are the most abundant archaeal phylum in the mangrove and mudflat sediments of Futian Nature Reserve (Shenzhen, China) and Mai Po Nature Reserve (Hong Kong, China) [6, 22]. Thus, following those studies, the total DNA and RNA of sediment samples from these two places were sequenced for constructing genomes and transcriptomes of Bathyarchaeota, respectively. Together with all available bathyarchaeotal MAGs in the public database (including the dozens of MAGs released lately [23]), we aimed to (1) search for the new metabolisms of Bathyarchaeota; (2) compare metabolic potentials among bathyarchaeotal subgroups; and (3) further predict the roles of Bathyarchaeota in the geochemical cycle.

## Results and discussion

### Genome construction and transcriptome

In total, eight layers in three sediment profiles from two habitats were selected for metagenomic and metatranscriptomic sequencing (Figure S1; details of the samples and sequencing are listed in Table S1). Raw DNA reads were trimmed, de novo assembled, and binned to obtain multiple MAGs. Among them, bathyarchaeotal MAGs were picked out and combined with reference bathyarchaeotal genomes to form a database, then short DNA reads of Bathyarchaeota were recovered by remapping DNA reads of all samples to the genome database. Finally, nine bathyarchaeotal MAGs were constructed by de novo assembling bathyarchaeotal reads and subsequent binning. All bathyarchaeotal MAGs ranged from ~ 0.6 to ~ 1.9 Mb in size, 34.68–58.90% G+C content, and estimated completeness (based on the presence of single-copy genes) of 58.03–95.33% (Table S2).

**Fig. 1:**
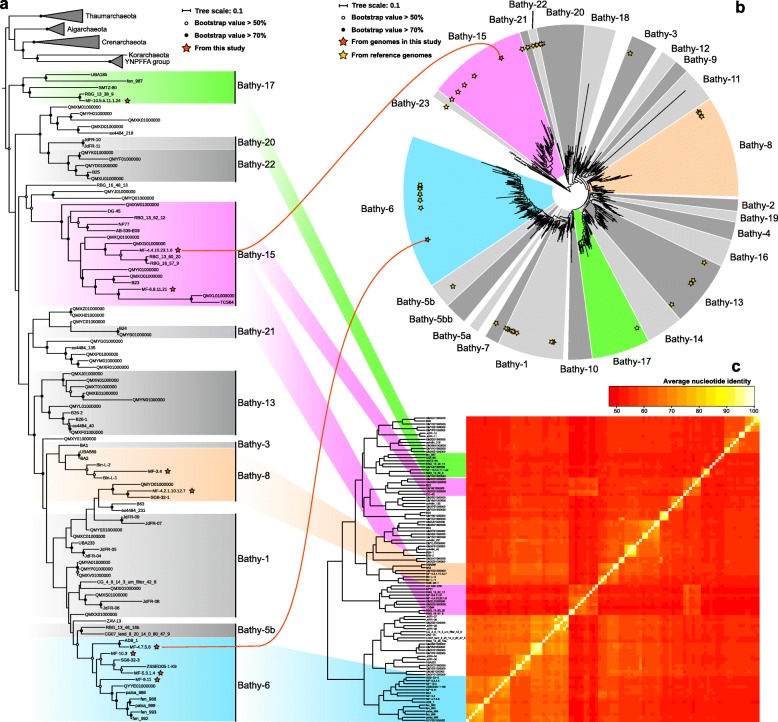
Subgroup assignment of bathyarchaeotal genomes by **a** phylogenetic tree based on 16 ribosomal proteins, **b** phylogenetic tree of 16S rRNA genes, and **c** average nucleotide identity of genomes. Two red lines represent two bathyarchaeotal MAGs with 16S rRNA genes in the current study. The scale bar indicates the average number of amino acid or nucleotide substitutions per site. The sequences were aligned independently using MUSCLE, columns with more than 95% gaps were trimmed using trimAL. The maximum likelihood trees of 16S rRNA gene and 16 ribosomal proteins were built using RAxML 8.0, the number of bootstraps was 1000, and the evolutionary models were GTRCAT (for 16S rRNA gene) and LG+GAMMA (for ribosomal protein), respectively

Phylogenetic analyses of 16 ribosomal proteins were conducted with all available bathyarchaeotal MAGs (91 reference genomes from database and 9 MAGs from this study; Fig. 1a) and high-completeness MAGs (containing all 16 ribosomal proteins; 22 reference genomes from database and 6 MAGs from this study; Figure S2), both of results show similar structure, confirming the valid subgroup assignments of bathyarchaeotal MAGs. Taken together with phylogenetic analysis of 16S rRNA genes and average nucleotide identity results (Fig. 1b, c), nine bathyarchaeotal MAGs in the current study were believed to belong to Bathy-6 (4 MAGs), -8 (2 MAGs), -15 (2 MAGs), and -17 (1 MAG), respectively. These four subgroups were also proved to be the major bathyarchaeotal subgroups in the previous reports of archaeal communities in both mangrove habitats [6, 22].

The coverages of metagenome and transcriptome to each MAG are shown in Figure S3 and Table S3. Similar to the bathyarchaeotal abundance in the mangrove and seafloor sediments using 16S rRNA gene sequencing [6, 18, 22], the metagenomic coverages of all MAGs were increased along with the sediment depth, with RPKM value from 0 (MF-5.3.1.4 in SZ_1) − 0.058 (MF-3.4 in SZ_1) in the surface to 0.017 (MF-10.5.5.11.1.24 in Maipo-9) − 0.392 (MF-3.4 in Maipo-9) in the deepest layer (Figure S3a and Table S3). However, the results of transcriptomic coverage had no significant correlations with depth, with the minimal coverage in SZ_2 (MF-10.5.5.11.1.24; RPKM value is 0) and maximal coverage in Maipo-8 (MF-9.11; RPKM value is 3.049) (Figure S3b and Table S3). These results suggested that genomic abundance of bathyarchaeotal members could not reflect their real transcritional activities in the sediments, and highlighted that it is important to investigate the transcriptome of the microbial community in the future ecological functions [24, 25].

### Light sensing

**Fig. 2:**
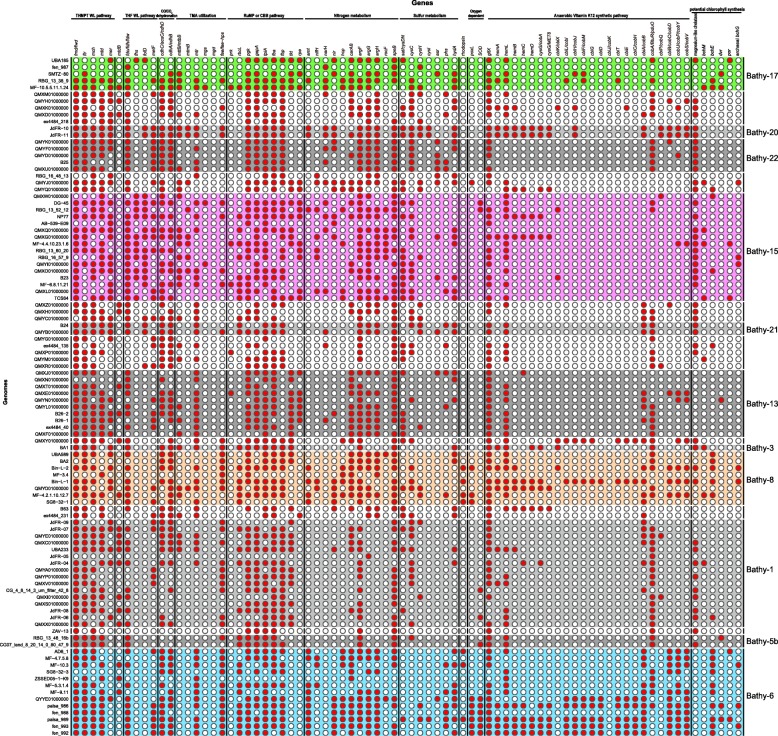
Presence (red) or absence (white) of marker genes within the metabolisms from each bathyarchaeotal genome

**Fig. 3:**
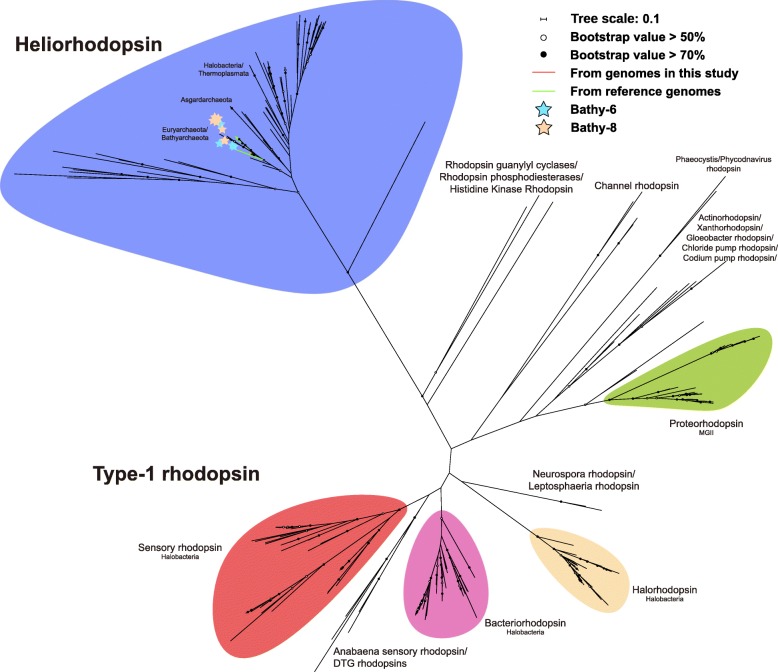
Maximum Likelihood tree of rhodopsin sequences. The scale bar indicates the average number of amino acid substitutions per site. The anchor sequences were from Pushkarev et al. [29]. Sequences were aligned using MUSCLE, columns with more than 95% gaps were trimmed using trimAL. The maximum likelihood tree was built using RAxML 8.0, the number of bootstraps was 1000, and the evolutionary model was LG+GAMMA

**Fig. 4:**
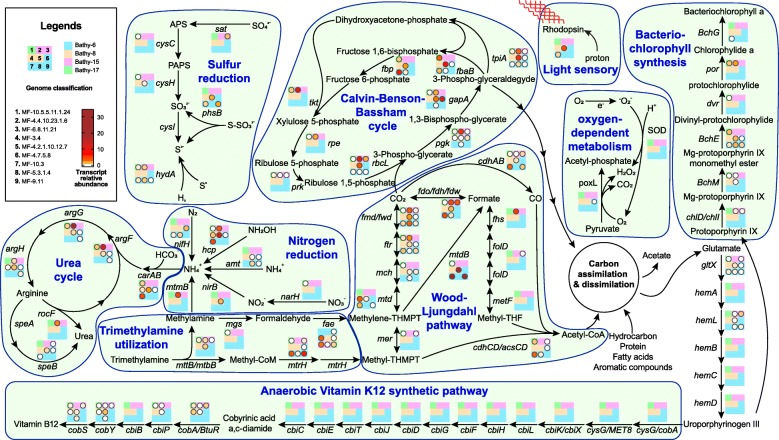
Metabolic pathways of nine bathyarchaeotal MAGs in the current study and the transcript activities of individual genes in each bin. Nine squares represent nine bathyarchaeotal MAGs in the current study, colors of the squares represent the subgroups MAGs belonged to, the absence of circles on the squares represents that the MAGs don’t harbor the gene, and the filled color of circles represents the transcript level of each gene normalized by ribosomal protein S3

Rhodopsins are membrane proteins engaged in light perception and are widespread in three domains of life. They are employed by many organisms to generate energy from light [26–28]. According to the annotation of bathyarchaeotal MAGs, rhodopsin genes were also found in the MAGs of Bathy-6 and -8 (Fig. 2). For further confirming the type of rhodopsin, a rhodopsin phylogenetic tree was constructed, clearly showing that the rhodopsins detected in Bathyarchaeota are heliorhodopsins (Fig. 3). Heliorhodopsins are newly described types of rhodopsins, which are abundant and globally distributed [29]. The photocycle of heliorhodopsins (including retinal isomerization and proton transfer, the same as in type-1 and type-2 rhodopsins) is long, which is common in sensory type-1 rhodopsins and benefits for the interaction between rhodopsins and transducer proteins [29]. This result suggests a light-sensory activity of heliorhodopsin, indicating that Bathyarchaeota may sense light. The metatranscriptomic analysis further supported the transcriptional activity for rhodopsin genes in Bathy-6 and -8 (Fig. 4), suggesting that members of Bathy-6 and -8 in mangrove sediments might sense light. However, previous studies have revealed that most of bathyarchaeotal members prefer subsurface of the sediments and large numbers of Bathyarchaeota were found in deeper biosphere where visible light could barely reach [6, 18, 22, 30], thus Bathyarchaeota may not capture visible light with rhodopsin. Infrared light has been proved to be an available energy source for some plants and bacteria [31–35], and rhodopsin could gain longer-wavelength or even infrared sensitivity by substituting all-trans-retinal (chromophore for archaeal cells) with 3,4-dehydroretinal [36], retinal A2, 3-methylamino-16-nor-1,2,3,4-didehydroretinal, or other analogs [37]. Previous studies have also shown that the retinal deficiency by deleting gene *sll1541* (converting carotenal to retinal) in bacterial cells could in vivo reconstitute far-red-absorbing rhodopsin with exogenous retinal analog (all-trans-3,4-dehydroretinal and 3-methylamino-16-nor-1,2,3,4-didehydroretinal) [38]. In the current study, two bathyarchaeotal MAGs were found to harbor the genes for carotenoid biosynthesis (*crtY*) and the genes encoding retinol dehydrogenase (RDH8, 11, 12, 13, 14) were identified in seven bathyarchaeotal MAGs (Table S4). It is possible that bathyarchaeotal cells may utilize exogenous retinal analogs and gain infrared energy. However, the genes *crtY* and RDH were not found in the same MAG, and the other essential genes for retinal biosynthesis (including the genes encoding carotene dioxygenase and retinoid isomerohydrolase) were still missing, thus more evidences were needed to support the utilization of retinal (or analogs) by Bathyarchaeota. Another possibility for bathyarchaeotal rhodopsin is that, Bathyarchaeota may orient themselves to the subsurface with the rhodopsin as photosensitive protein. The genes for flagella biosynthesis were widespread in bathyarchaeotal MAGs, which is in agreement with the previous report [15], suggesting that bathyarchaeotal cells are capable of motion. Since rhodopsin could response to light by delivering electron, fading, or even breaking down [29, 39], the light sensory of Bathyarchaeota may possibly be one of forces to drive them towards the suitable habitats in subsurface sediments. However, more additional works are needed to tell the importance of bathyarchaeotal rhodopsin.

In addition, by searching for the rhodopsin genes in archaeal genomes, plenty of archaeal rhodopsin sequences were found, and phylogenetic analysis implied that heliorhodopsin genes were also harbored by many archaeal phyla, including Euryarchaeota and Asgard archaea (Fig. 3), suggesting heliorhodopsin may be a common protein for archaea to perceive light [40].

### Porphyrin biosynthesis

Porphyrin is an important type of tetrapyrrole for living organisms on Earth, many biological processes, including photosynthesis, respiration, circulation, and nutrition, are dependent on the compounds derived from it (chlorophylls, coenzyme F_430_, hemes, and cobalamin, respectively) [41, 42]. The biosynthesis of these derived compounds all starts with synthesizing Uroporphyrinogen III from glutamate or glycine, then different metal ions are chelated in porphyrin rings by different chelatases, in which dozens of enzymes are involved [43]. In the current study, all of genes related to anaerobic cobalamin biosynthesis were found in Bathyarchaeota, and some members within bathy-6, -8, and -20 were found to harbor more than half of them (including cobalt chelatase *cbiK* and *cbiX*), suggesting the potential cobalamin biosynthesis by Bathyarchaeota (Fig. 2). Cobalamin, also named Vitamin B12, is an essential enzyme cofactor in DNA, fatty acid, and amino acid metabolisms for all lives [44]. Cobalamin can only be produced in nature by a few bacteria and archaea [45], thus eukaryotic organisms and cobalamin auxotrophic microbes rely on them. A previous study suggests some members within domain Archaea serve as cobalamin producers in natural environments, including Euryarchaeota and Thaumarchaeota [44, 46–48]. To our knowledge, this is the first report to provide the genetic evidence of cobalamin biosynthetic pathway in two subgroups of Bathyarchaeota. This finding suggests that some members of Bathyarchaeota may benefit the growth of other lives via vitamin B12 production in diverse environments.

Interestingly, the phylogenetic analysis of the chelatase genes in Bathyarchaeota indicated that, besides cobalt chelatase (*cbiK* and *cbiX*), many magnesium chelatase genes were also harbored by Bathyarchaeota (cluster with *chlD* and *chlI*) (Figure S4), and most of bathyarchaeotal MAGs with the magnesium chelatase genes (including members of Bathy-1, -3, -15, -20, and -22) did not harbor the genes for cobalamin biosynthesis (Fig. 2). Magnesium chelatase is known to work in the first unique step of (bacterio)chlorophyll biosynthesis by inserting magnesium ion into protoporphyrin IX [49], further gene exploring indicated that some genes related to chlorophyll synthesis are also found in bathyarchaeotal MAGs (Bathy-6, -8, -15, and -17 in Fig. 2), thus the existence of magnesium chelatase genes might support a potential chlorophyll biosynthesis, suggesting the metabolic diversity in Bathyarchaeota.

### Calvin-Benson-Bassham (CBB) cycle

**Fig. 5:**
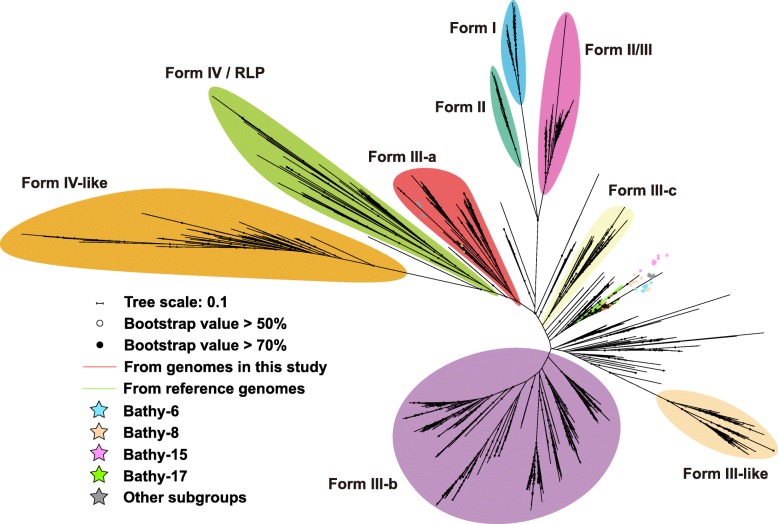
Maximum Likelihood tree of RuBisCO sequences. The scale bar indicates the average number of amino acid substitutions per site. The anchor sequences were from Jaffe et al. [51]. Sequences were aligned using MUSCLE, columns with more than 95% gaps were trimmed using trimAL. The maximum likelihood tree was built using RAxML 8.0, the number of bootstraps was 1000, and the evolutionary model was LG+GAMMA

Ribulose-1,5-bisphosphate carboxylase/oxygenase (RuBisCO) and phosphoribulokinase (PRK) are two representative enzymes of the CBB cycle [50]. In the current study, among 100 available bathyarchaeotal genomes, 33 genomes within 8 subgroups, including Bathy-6, -8, -15, and -17, harbored the genes of RuBisCO (Fig. 2), and they phylogenetically belonged to Form III (including both Forms III-a and III-b) (Fig. 5). The genes of PRK were found in the genomic bins of Bathy-15 and -17 (Fig. 2 and Figure S5), with transcript activity in Bathy-17 (Fig. 4). In comparison with the short scaffolds harboring the genes of PRK, some RuBisCO genes were harbored by the long scaffolds (> 10 kbp) encoding ribosomal proteins (in genomes B24, SG8-32-3, MF-5.3.1.4, etc.) and other CBB cycle-related enzymes (in genomes BA1, MF-10.3, MF-4.2.1.10.12.7, etc.), further supporting that Bathyarchaeota may participate in CBB cycle. Notably, it is the first time to report a Form III-a RuBisCO in bathyarchaeotal MAGs. Previously, Form III-a RuBisCO has only been identified in methanogens [51], which employ both PRK and Form III-a RuBisCO to regenerate carbon fixation [52]. A previous study has demonstrated that even *Escherichia coli* could generate a functional CBB cycle with the co-existence of RuBisCO and PRK [53]. Thus, considering that bathyarchaeotal MAGs harbored all genes of CBB cycle, including RuBisCO, *prk*, phosphoglycerate kinase (*pgk*), glyceraldehyde-3-phosphate dehydrogenase (*gapA*), triosephosphate isomerase (*tpiA*), fructose-bisphosphate aldolase (*fbaB*), fructose-1,6-bisphosphatase (*fbp*), transketolase (*tkt*), and ribulose-phosphate 3-epimerase (*rpe*), and they all have transcript activities (MF−10.5.5.11.1.24 in Fig. 4), all of the results suggested the metabolic potential for carbon fixation through the CBB cycle in the bathyarchaeotal cells. Taken together with the potential chlorophyll biosynthesis pathway described above, members of Bathyarchaeota may possess both metabolic pathways for carbon fixation and light sense (potential chlorophyll based and/or rhodopsin based). However, the co-existence and relationship of these two pathways in Bathyarchaeota are unknown, more works are needed to verify.

### Nitrogen metabolism

Several studies have found genomic evidence that Bathyarchaeota are involved in the nitrogen cycle [13, 15, 54]. In the current study, more nitrogen-related genes, including ammonium transporter (*amt*), hydroxylamine reductase (*hcp*), respiratory nitrate reductase (*narH*), nitrite reductase (*nir*), nitrogenase iron protein (*nifH*), and mono/di/trimethylamine aminotransferase (*mttB*/*mtbB*/*mtmB*), were found in bathyarchaeotal MAGs, and different bathyarchaeotal subgroups harbored different ones (Fig. 2). Taken together with the different transcript activities of these genes in different subgroups (Fig. 4), bathyarchaeotal members may be capable of producing ammonium with diverse nitrogen compounds. Genes involving in urea production were also found in bathyarchaeotal MAGs (Fig. 2) with high transcriptional activities (Fig. 4), further suggesting that Bathyarchaeota may convert ammonium to urea. For life in the ocean, nitrogen is a limiting nutrient [55], and the current study suggests that Bathyarchaeota may utilize diverse primary nitrogen sources to produce urea (Fig. 4), suggesting that Bathyarchaeota may act as a “transfer station” for nitrogen compounds in the global nitrogen cycle.

Moreover, for urea producing, two pathways, including arginase (*rocF*) and agmatinase (*speB*) pathways, were both found in Bathyarchaeota. Different from the widespread of *speB* in all bathyarchaeotal subgroups, *rocF* only existed in the MAGs of Bathy-6, -8 and -15 (Fig. 2), and had transcriptional activity only in Bathy-15 (Fig. 4). Gene *rocF* is formerly known only existing in the members of bacteria and eukaryotes [56]; however, according to the phylogenetic analysis in the current study, in addition to Bathyarchaeota, *rocF* genes were also found existing in Woesearchaeota and Thorarchaeota, and they formed a distinct clade in the phylogenetic tree (Figure S6), indicating that archaeal arginase evolves independently from those of Bacteria and Eukaryotes.

### Sulfur metabolism

Sulfate or sulfite was previously reported as the important environmental factors to shape the distribution of bathyarchaeotal subgroups [18, 30, 57], and genomic evidence for dissimilatory sulfate and sulfite reduction via genes *sat*-*aprAB* (sulfate adenylyltransferase-adenylylsulfate reductase) were also reported [17, 58]. They both suggested that Bathyarchaeota could participate the global sulfur cycle. In the current study, different from previous studies, diverse genes related to assimilatory sulfur reduction via genes *cysND-cysC-cysH-cysI* (sulfate adenylyltransferase-phosphoadenosine phosphosulfate reductase-sulfite reductase) were identified from the bathyarchaeotal genomes (Fig. 2). Similar to the nitrogen metabolism, different subgroups of Bathyarchaeota harbored parts of sulfur reducing metabolism: more than half of genomes within Bathy-15 and -17 harbored the genes related to sulfate reduction (*cysND*, *cysC*, and *cycH*), while the gene *cysI* only detected in one Bathy-6 genome, and most of the genomes within Bathy-6 harbored the gene related to thiosulfate reduction (*phs*) (Fig. 2). The transcriptional activities of the genes within each subgroup were also different from each other (Fig. 4), suggesting different subgroups of Bathyarchaeota may participate in different parts of the sulfur cycle. In addition, most members of Bathyarchaeota may have the ability to reduce S^0^ to sulfide with *hydA* (hydrogenase/sulfur reductase), supporting the previous studies that high abundance of Bathyarchaeota in the sulfur-rich habitats [12, 20, 59, 60]. All of these results indicated a role of Bathyarchaeota in the global sulfur cycle.

### Distinct microoxic lifestyle of Bathy-6

Notably, the genes related to the oxygen-dependent pathways were found in bathyarchaeotal MAGs, including pyruvate oxidase (*poxL*) in Bathy-6 and -8, and superoxide dismutase (SOD) in Bathy-1, -6, and -15 (Fig. 2 and Figure S7). In particular, most MAGs of Bathy-6 did not harbor *poxL* and SOD genes, while six reference MAGs within Bathy-6 harbor both genes (Fig. 2), suggesting that some members of Bathy-6 may live aerobically. Further, the phylogenetic analysis of bathyarchaeotal MAGs indicated that, the MAGs harboring the genes of both cobalamin biosynthesis (more than half of related genes) and oxygen-dependent pathways were phylogenetically clustered together and formed a functionally distinctive lineage within Bathy-6 (Figs. 1 and 2). In addition, rhodopsin was also found in the MAGs within this lineage, suggesting that members of this lineage may be a source of vitamin B12 preferring microoxic habitats with/without accessible light. It is totally different from the anoxic lifestyle of the other bathyarchaeotal members, supporting the distinct niche preference of Bathy-6 in the previous study [22, 30] and suggesting versatile metabolic abilities and varied lifestyles within Bathy-6.

## Conclusions

Previous genomic analyses have suggested that Bathyarchaeota was an important driver for global carbon cycle. However, many potential metabolisms are ignored, thus it is underestimating the importance of Bathyarchaeota in global biochemical cycle. In this study, Bathyarchaeota was firstly found to potentially involve in rhodopsin and porphyrin biosynthesis, CBB cycle, and some pathways related to nitrogen and sulfur cycles. The potential biosynthetic pathway of rhodopsin and chlorophyll-like compounds suggested phototrophy of Bathyarchaeota, the potential biosynthesis of cobalamin indicated a possible vitamin B12 production by some Bathyarchaeota, and the pathway of utilizing diverse nitrogen compounds to produce urea implied that Bathyarchaeota might be an important “transfer station” for marine nitrogen cycle. Moreover, some members of Bathy-6 were found to have a light-sensory, vitamin B12 producing, and microoxic lifestyle, highlighting diverse metabolic abilities among or even within bathyarchaeotal subgroups. Considering Bathyarchaeota is a widespread and high-abundance phylum in diverse environments, the new knowledges of bathyarchaeotal metabolisms in the current study further highlight the crucial role of Bathyarchaeota in the global biochemical cycle.

## Methods

### Sample collection, DNA and RNA extraction, and sequencing

Mangrove wetland often occurs in subtropical coastal regions, and it supports plenty of plants, animals, meio/macro-fauna, and prokaryotes, contributes up to 15% of all carbon accumulation in marine settings [61, 62]. Futian Nature Reserve (Shenzhen, China) and Mai Po Nature Reserve (Hong Kong, China) are located at the north and south sides of Shenzhen Bay in Southern of China, respectively, and their mangrove forests join at the estuarine mouth of Shenzhen River (Figure S1). As described in the recent studies [13, 63], sediment cores were collected from the mangrove and mudflat in Futian Nature Reserve (Shenzhen, China) and Mai Po Nature Reserve (Hong Kong, China) using columnar samplers (Figure S1). Eight samples were picked out and put in an icebox before taken to the lab. Samples for RNA extraction were preserved in RNAlater (Ambion, China). For each sample, 10 g sediment was used for DNA and RNA extraction with PowerSoil DNA Isolation Kit and RNA Powersoil Total RNA Isolation Kit (QIAGEN, German), respectively. For RNA samples, Ribo-Zero rRNA removal kit (Illumina, USA) was used to remove rRNA, and the reverse transcription of remaining RNA was conducted using SuperScript III First Strand Synthesis System (Invitrogen, USA). Consequently, DNA and cDNA were sequenced using Illumina HiSeq 4000 (USA) PE150 by BerryGenomics (China).

### Metagenome assembly, genome binning, and gene annotation

Raw metagenomic reads were dereplicated (100% identity over 100% length) and trimmed using sickle [64]. Remaining reads of each sample were de novo assembled using IDBA-UD [65] with the parameters -mink 65, -maxk 145, and -steps 10. The binning of scaffolds was conducted using MetaBAT [66] with 12 sets of parameters. Then, 12 results were analyzed using Das Tool [67] to obtain the optimized genomic bins. To improve the qualities of the bins, the scaffolds of bathyarchaeotal bins and reference genomes were remapped by the raw reads of all samples using BWA [68], all mapped reads were repeated assembling and binning as above. Finally, the genomic bins were decontaminated based on the results of contig-cluster tree using anvio5 (http://merenlab.org/software/#anvio). The completeness and contamination of MAGs were calculated using CheckM [69]. The taxonomic assignment of the MAGs was conducted with GTDB-Tk package [70] to ensure them belonging to Bathyarchaeota (Table S5), subgroup assignment was performed by building phylogenetic trees (see “Phylogenetic analyses and average nucleotide identity” section).

16S rRNA genes were predicted and taxonomically assigned by BLASTn against the SILVA NR99 database (v132) [71]. Genes were called using Prodigal with parameter -p meta [72]. Genes were annotated using KEGG Automatic Annotation Server [69] and BLASTp against NR database retrieved on December 2017 (*e* value < 1e−5). To further confirm the annotation of the marker genes related to Calvin-Benson-Bassham (CBB) cycle, urea cycle, light sensing, porphyrin biosynthesis, and microoxic lifestyle, amino acid sequences of ribulose-1,5-bisphosphate carboxylase/oxygenase (RuBisCO), phosphoribulokinase (PRK), arginase/agmatinase, rhodopsin, chelatase, and superoxide dismutase (SOD) were downloaded from UniProt database (Accessed July 2019) [73] to form the local ones, and the amino acid sequences called from bathyarchaeotal MAGs were BLASTp against the local database (*e* value < 1e−5). Finally, phylogenetic trees were built to ensure the annotation of the genes. Details of the related gene annotation are shown in Table S4.

### Metagenomic and transcriptomic abundance of sequences

The gene abundance from each MAG was determined by mapping metagenomic reads to the sequences using BWA software with the default setting [68], and the relative abundances were calculated using the RPKM method [74]. Transcript abundance of predicted genes was calculated by mapping non-rRNA transcriptomic reads to gene sequences as above, and the relative abundance of each gene was normalized by the abundance of ribosomal protein S3, considering its transcripts could be detected in all bathyarchaeotal MAGs as single-copy conserved gene. Details of transcript level of the predicted genes are shown in Table S6.

### Phylogenetic analyses and average nucleotide identity

Phylogenetic tree of 16S rRNA gene was built with all 16S rRNA gene sequences from bathyarchaeotal MAGs and the reference sequences from Zhou et al. [13]. Phylogenetic analysis of genomes was conducted with 16 ribosomal protein data sets (ribosomal proteins L2, L3, L4, L5, L6, L14, L15, L16, L18, L22, L24, S3, S8, S10, S17, and S19) [75] predicted by CheckM [69]. The phylogenetic trees of the functional proteins were built with sequences from the MAGs and anchor sequences from Jaffe et al. [51] (RuBisCO and PRK), Pushkarev et al. [29] (rhodopsin), Novák et al. [76] (agmatinase and arginase), or the sequences of local database mentioned above (chelatase and SOD), respectively. All trees were constructed as below: sequences were aligned independently using MUSCLE [77], columns with more than 95% gaps were trimmed using trimAL [78]. Before building tree, 16 ribosomal protein alignments were concatenated, and the taxa with less than 50% of the alignment columns were removed. The maximum likelihood trees of 16S rRNA gene, 16 ribosomal proteins, and functional proteins were built using RAxML 8.0 [79] on the CIPRES Science Gateway [80], the number of bootstraps was 1000, and the evolutionary models were GTRCAT (for nucleotide) and LG+GAMMA (for amino acid), respectively. Then, the trees were visualized on the iTOL web server [81].

The pairwise average nucleotide identity between each bathyarchaeotal genome was calculated and plotted by using get_homologues package [82] with default parameters.

## Supplementary information

**Additional file 1: Table S1.** Information and sequencing details of all samples.

**Additional file 2: Table S2.** Detail information of bathyarchaeotal genomic bins.

**Additional file 3: Table S3.** Metagenomic and transcriptomic coverage of bathyarchaeotal genomic bins.

**Additional file 4: Table S4.** Annotated results of the genes mentioned in the current study.

**Additional file 5: Table S5.** Taxonomic assignment results of bathyarchaeotal genomic bins using GTDB-Tk package.

**Additional file 6: Table S6.** Transcript value (normalized by RPKM method) of the genes in each genomic bin.

**Additional file 7: Figure S1.** The location of the sample sites and the depths of the samples.

**Additional file 8: Figure S2.** Subgroup assignment and phylogenetic tree of bathyarchaeotal genomes containing all 16 ribosomal proteins.

**Additional file 9: Figure S3.** The metagenomic and transcriptomic coverages of nine bathyarchaeotal genomes in this study.

**Additional file 10: Figure S4.** Maximum Likelihood tree of chelatase sequences. The scale bar indicates the average number of amino acid substitutions per site. The anchor sequences and methods are in Materials and methods.

**Additional file 11: Figure S5.** Maximum Likelihood tree of PRK sequences. The scale bar indicates the average number of amino acid substitutions per site. The anchor sequences and methods are in Materials and methods.

**Additional file 12: Figure S6.** Maximum Likelihood tree of arginase and agmatinase sequences. The scale bar indicates the average number of amino acid substitutions per site. The anchor sequences and methods are in Materials and methods.

**Additional file 13: Figure S7.** Maximum Likelihood tree of SOD sequences. The scale bar indicates the average number of amino acid substitutions per site. The anchor sequences and methods are in Materials and methods.

**Additional file 14: Supplementary data 1.** The explanation for gene abbreviations using in Fig. 2 and 4, Table S4, and S6.

**Additional file 15: Supplementary data 2.** The multiple amino acid alignment of rhodopsin sequences using in Fig. 3.

**Additional file 16: Supplementary data 3.** The multiple amino acid alignment of RuBisCO sequences using in Fig. 5.

**Additional file 17: Supplementary data 4.** The multiple amino acid alignment of chelatase sequences using in Figure S4.

**Additional file 18: Supplementary data 5.** The multiple amino acid alignment of phosphoribulokinase sequences using in Figure S5.

**Additional file 19: Supplementary data 6.** The multiple amino acid alignment of arginase and agmatinase sequences using in Figure S6.

**Additional file 20: Supplementary data 7.** The multiple amino acid alignment of SOD sequences using in Figure S7.

## Data Availability

The metagenome and transcriptome data generated during the current study are available in NCBI database under the project number PRJNA360036. The data of Bathyarchaeota MAGs analyzed during the current study are available in NCBI database under the accession numbers SMYP00000000-SMYX00000000.

## Abbreviations

*amt*: Ammonium transporter
CBB: Calvin-Benson-Bassham
*cbiK*/*cbiX*: Sirohydrochlorin cobaltochelatase
*chlD*: Magnesium-chelatase subunit ChlD
*chlI*: Magnesium-chelatase subunit ChlI
*crtY*: Carotenoid biosynthesis
*fbaB*: Fructose-bisphosphate aldolase
*fbp*: Fructose-16-bisphosphatase
*gapA*: Glyceraldehyde-3-phosphate dehydrogenase
*hcp*: Hydroxylamine reductase
*hydA*: Hydrogenase/sulfur reductase
MAG: Metagenome-assembled genome
*mtbB*: Dimethylamine aminotransferase
*mtmB*: Monomethylamine aminotransferase
*mttB*: Trimethylamine aminotransferase
*narH*: Respiratory nitrate reductase
*nifH*: Nitrogenase iron protein
*nir*: Nitrite reductase
*pgk*: Phosphoglycerate kinase
*poxL*: Pyruvate oxidase
PRK: Phosphoribulokinase
RDH: Retinol dehydrogenase
*rocF*: Arginase
*rpe*: Ribulose-phosphate 3-epimerase
RuBisCO: Ribulose-15-bisphosphate carboxylase/oxygenase
SAG: Single-cell genome
SOD: Superoxide dismutase
*speB*: Agmatinase
*tkt*: Transketolase
*tpiA*: Triosephosphate isomerase

## References

[1] Inagaki F, Suzuki M, Takai K, Oida H, Sakamoto T, Aoki K (2003). Microbial communities associated with geological horizons in coastal subseafloor sediments from the sea of okhotsk. Appl Environ Microbiol..

[2] Meng J, Xu J, Qin D, He Y, Xiao X, Wang F (2014). Genetic and functional properties of uncultivated MCG archaea assessed by metagenome and gene expression analyses. ISME J..

[3] Lloyd KG, Schreiber L, Petersen DG, Kjeldsen KU, Lever MA, Steen AD (2013). Predominant archaea in marine sediments degrade detrital proteins. Nature..

[4] Petitjean C, Deschamps P, López-García P, Moreira D (2014). Rooting the domain archaea by phylogenomic analysis supports the foundation of the new kingdom Proteoarchaeota. Genome Biol Evol..

[5] Takai S, Henton MM, Picard JA, Guthrie AJ, Fukushi H, Sugimoto C (2001). Prevalence of virulent Rhodococcus equi in isolates from soil collected from two horse farms in South Africa and restriction fragment length polymorphisms of virulence plasmids in the isolates from infected foals, a dog and a monkey. Onderstepoort J Vet Res..

[6] Zhou Z, Meng H, Liu Y, Gu J-D, Li M (2017). Stratified bacterial and archaeal community in mangrove and intertidal wetland mudflats revealed by high throughput 16S rRNA gene sequencing. Front Microbio..

[7] Vaksmaa A, van Alen TA, Ettwig KF, Lupotto E, Vale G, Jetten MSM (2017). Stratification of diversity and activity of methanogenic and methanotrophic microorganisms in a nitrogen-fertilized Italian paddy soil. Front Microbiol..

[8] Xia X, Guo W, Liu H (2017). Basin scale variation on the composition and diversity of archaea in the pacific ocean. Front Microbiol..

[9] Inagaki F, Nunoura T, Nakagawa S, Teske A, Lever M, Lauer A (2006). Biogeographical distribution and diversity of microbes in methane hydrate-bearing deep marine sediments, on the Pacific Ocean Margin. Proc Natl Acad Sci U S A..

[10] Seyler LM, McGuinness LM, Kerkhof LJ (2014). Crenarchaeal heterotrophy in salt marsh sediments. ISME J..

[11] Li M, Jain S, Dick GJ (2016). Genomic and Transcriptomic Resolution of Organic Matter Utilization Among Deep-Sea Bacteria in Guaymas Basin Hydrothermal Plumes. Front Microbiol..

[12] Kubo K, Lloyd KG, Biddle JF, Amann R, Teske A, Knittel K (2012). Archaea of the miscellaneous crenarchaeotal group are abundant, diverse and widespread in marine sediments. ISME J..

[13] Zhou Z, Pan J, Wang F, Gu JD, Li M (2018). Bathyarchaeota: globally distributed metabolic generalists in anoxic environments. FEMS Microbiol Rev..

[14] Biddle JF, Lipp JS, Lever MA, Lloyd KG, Sorensen KB, Anderson R (2006). Heterotrophic Archaea dominate sedimentary subsurface ecosystems off Peru. Proc Natl Acad Sci U S A..

[15] Lazar CS, Baker BJ, Seitz K, Hyde AS, Dick GJ, Hinrichs KU (2016). Genomic evidence for distinct carbon substrate preferences and ecological niches of Bathyarchaeota in estuarine sediments. Environ Microbiol..

[16] He Y, Li M, Perumal V, Feng X, Fang J, Xie J (2016). Genomic and enzymatic evidence for acetogenesis among multiple lineages of the archaeal phylum Bathyarchaeota widespread in marine sediments. Nat Microbiol..

[17] Evans PN, Parks DH, Chadwick GL, Robbins SJ, Orphan VJ, Golding SD (2015). Methane metabolism in the archaeal phylum Bathyarchaeota revealed by genome-centric metagenomics. Science..

[18] Yu T, Liang Q, Niu M, Wang F (2017). High occurrence of Bathyarchaeota (MCG) in the deep-sea sediments of South China Sea quantified using newly designed PCR primers. Environ Microbiol Rep..

[19] Fillol M, Auguet JC, Casamayor EO, Borrego CM (2016). Insights in the ecology and evolutionary history of the Miscellaneous Crenarchaeotic Group lineage. ISME J..

[20] Xiang X, Wang RC, Wang HM, Gong LF, Man BY, Xu Y (2017). Distribution of Bathyarchaeota communities across different terrestrial settings and their potential ecological functions. Sci Rep.

[21] Zou D, Pan J, Liu Z, Zhang C, Liu H, Li M. The distribution of Bathyarchaeota in surface sediments of the Pearl river estuary along salinity gradient. Front Microbio. 2020. 10.3389/fmicb.2020.00285. 10.3389/fmicb.2020.00285 PMC7056671 32174899

[22] Pan J, Chen Y, Wang Y, Zhou Z, Li M (2019). Vertical distribution of Bathyarchaeotal communities in mangrove wetlands suggests distinct niche preference of Bathyarchaeota subgroup 6. Microb Ecol..

[23] Tully BJ, Graham ED, Heidelberg JF (2018). The reconstruction of 2,631 draft metagenome-assembled genomes from the global oceans. Sci Data..

[24] Chen L-X, Hu M, Huang L-N, Hua Z-S, Kuang J-L, Li S-J (2014). Comparative metagenomic and metatranscriptomic analyses of microbial communities in acid mine drainage. ISME J.

[25] Lee SW, Kuan CS, Wu LS, Weng JT (2016). Metagenome and metatranscriptome profiling of moderate and severe COPD sputum in Taiwanese Han Males. PLoS One..

[26] Beja O, Spudich EN, Spudich JL, Leclerc M, DeLong EF (2001). Proteorhodopsin phototrophy in the ocean. Nature..

[27] Finkel OM, Beja O, Belkin S (2013). Global abundance of microbial rhodopsins. ISME J..

[28] Slamovits CH, Okamoto N, Burri L, James ER, Keeling PJ (2011). A bacterial proteorhodopsin proton pump in marine eukaryotes. Nat Commun..

[29] Pushkarev A, Inoue K, Larom S, Flores-Uribe J, Singh M, Konno M (2018). A distinct abundant group of microbial rhodopsins discovered using functional metagenomics. Nature..

[30] Lazar CS, Biddle JF, Meador TB, Blair N, Hinrichs KU, Teske AP (2015). Environmental controls on intragroup diversity of the uncultured benthic archaea of the miscellaneous Crenarchaeotal group lineage naturally enriched in anoxic sediments of the White Oak River estuary (North Carolina, USA). Environ Microbiol..

[31] Larkum AWD, Ritchie RJ, Raven JA (2018). Living off the Sun: chlorophylls, bacteriochlorophylls and rhodopsins. Photosynthetica..

[32] Antal T, Harju E, Pihlgren L, Lastusaari M, Tyystjärvi T, Hölsä J (2012). Use of near-infrared radiation for oxygenic photosynthesis via photon up-conversion. Int J Hydrogen Energy..

[33] Ritchie RJ, Larkum AWD, Ribas I (2018). Could photosynthesis function on Proxima Centauri b?. Int J Astrobiol.

[34] Shanmugam S, Xu J, Boyer C (2016). Light-regulated polymerization under near-infrared/far-red irradiation catalyzed by bacteriochlorophylla. Angew Chem.

[35] Oshita K, Suzuki T, Kawano T (2018). Possible roles of near-infrared light on the photosynthesis in *Synechocystis* sp. PCC6803 under solar simulating artificial light. Enviro Control Biol.

[36] Sineshchekov OA, Govorunova EG, Wang J, Spudich JL (2012). Enhancement of the long-wavelength sensitivity of optogenetic microbial rhodopsins by 3,4-dehydroretinal. Biochemistry..

[37] Ganapathy S, Kratz S, Chen Q, Hellingwerf KJ, de Groot HJM, Rothschild KJ (2019). Redshifted and near-infrared active analog pigments based upon archaerhodopsin-3. Photochem Photobiol.

[38] Chen Q, van der Steen JB, Arents JC, Hartog AF, Ganapathy S, de Grip WJ (2018). Deletion of *sll1541* in *Synechocystis* sp. Strain PCC 6803 Allows Formation of a Far-Red-Shifted *holo*-Proteorhodopsin *In Vivo*. Appl Environ Microbiol..

[39] Hubbard R (1958). Bleaching of Rhodopsin by light and by heat. Nature.

[40] Bulzu P-A, Andrei A-Ş, Salcher MM, Mehrshad M, Inoue K, Kandori H, et al. Casting light on Asgardarchaeota metabolism in a sunlit microoxic niche. Nat Microbiol. 2019. 10.1038/s41564-019-0404-y. 10.1038/s41564-019-0404-y 30936485

[41] Frankenberg N, Moser J, Jahn D (2003). Bacterial heme biosynthesis and its biotechnological application. Appl Microbiol Biotechnol..

[42] Battersby AR (2000). Tetrapyrroles: the pigments of life. Nat Prod Rep..

[43] Mauzerall DC (1998). Evolution of porphyrins. Clin Dermatol..

[44] Doxey AC, Kurtz DA, Lynch MD, Sauder LA, Neufeld JD (2015). Aquatic metagenomes implicate Thaumarchaeota in global cobalamin production. ISME J..

[45] Fang H, Kang J, Zhang D (2017). Microbial production of vitamin B12: a review and future perspectives. Microb Cell Fact..

[46] Escalante-Semerena JC (2007). Conversion of cobinamide into adenosylcobamide in bacteria and archaea. J Bacteriol..

[47] Fang H, Kang J, Zhang D (2017). Microbial production of vitamin B(12): a review and future perspectives. Microb Cell Fact.

[48] Woodson JD, Reynolds AA, Escalante-Semerena JC (2005). ABC transporter for corrinoids in Halobacterium sp. strain NRC-1. J Bacteriol..

[49] Martin WF, Sousa FL, Shavit-Grievink L, Allen JF (2012). Chlorophyll biosynthesis gene evolution indicates photosystem gene duplication, not photosystem merger, at the origin of oxygenic photosynthesis. Genome Biol Evol..

[50] Raines CA (2003). The Calvin cycle revisited. Photosynth Res..

[51] Jaffe AL, Castelle CJ, Dupont CL, Banfield JF (2018). Lateral gene transfer shapes the distribution of RuBisCO among candidate phyla radiation bacteria and DPANN archaea. Mol Biol Evol..

[52] Kono T, Mehrotra S, Endo C, Kizu N, Matusda M, Kimura H (2017). A RuBisCO-mediated carbon metabolic pathway in methanogenic archaea. Nat Commun..

[53] Antonovsky N, Gleizer S, Noor E, Zohar Y, Herz E, Barenholz U (2016). Sugar synthesis from CO2 in Escherichia coli. Cell..

[54] Harris RL, Lau MCY, Cadar A, Bartlett DH, Cason E, van Heerden E, et al. Draft Genome Sequence of “*Candidatus Bathyarchaeota*” Archaeon BE326-BA-RLH, an Uncultured Denitrifier and Putative Anaerobic Methanotroph from South Africa’s Deep Continental Biosphere. Microbiol Resour Announc. 2018;7. 10.1128/MRA.01295-18. 10.1128/MRA.01295-18 PMC6256629 30533830

[55] Bristow LA, Mohr W, Ahmerkamp S, Kuypers MMM (2017). Nutrients that limit growth in the ocean. Curr Biol..

[56] Wagemaker MJ, Welboren W, van der Drift C, Jetten MS, Van Griensven LJ, Op den Camp HJ (2005). The ornithine cycle enzyme arginase from Agaricus bisporus and its role in urea accumulation in fruit bodies. Biochim Biophys Acta.

[57] Fillol M, Sanchez-Melsio A, Gich F, Borrego CM (2015). Diversity of Miscellaneous Crenarchaeotic Group archaea in freshwater karstic lakes and their segregation between planktonic and sediment habitats. FEMS Microbiol Ecol.

[58] Zhang W, Ding W, Yang B, Tian R, Gu S, Luo H (2016). Genomic and transcriptomic evidence for carbohydrate consumption among microorganisms in a cold seep brine pool. Front Microbiol..

[59] Dahle H, Okland I, Thorseth IH, Pederesen RB, Steen IH (2015). Energy landscapes shape microbial communities in hydrothermal systems on the Arctic Mid-Ocean Ridge. ISME J..

[60] Barns SM, Delwiche CF, Palmer JD, Pace NR (1996). Perspectives on archaeal diversity, thermophily and monophyly from environmental rRNA sequences. Proc Natl Acad Sci U S A..

[61] Duarte CM, Middelburg JJ, Caraco N (2005). Major role of marine vegetation on the oceanic carbon cycle. Biogeosciences.

[62] Jennerjahn TC, Ittekkot V (2002). Relevance of mangroves for the production and deposition of organic matter along tropical continental margins. Naturwissenschaften..

[63] Cai M, Liu Y, Zhou Z, Yang Y, Pan J, Gu J-D, et al. Asgard archaea are diverse, ubiquitous, and transcriptionally active microbes. bioRxiv. 2018.

[64] Joshi N, Fass J (2011). Sickle: a sliding-window, adaptive, quality-based trimming tool for FastQ files.

[65] Peng Y, Leung HC, Yiu SM, Chin FY (2012). IDBA-UD: a de novo assembler for single-cell and metagenomic sequencing data with highly uneven depth. Bioinformatics..

[66] Kang DD, Froula J, Egan R, Wang Z (2015). MetaBAT, an efficient tool for accurately reconstructing single genomes from complex microbial communities. PeerJ..

[67] Sieber CMK, Probst AJ, Sharrar A, Thomas BC, Hess M, Tringe SG (2018). Recovery of genomes from metagenomes via a dereplication, aggregation and scoring strategy. Nat Microbiol..

[68] Li H, Durbin R (2009). Fast and accurate short read alignment with Burrows-Wheeler transform. Bioinformatics..

[69] Parks DH, Imelfort M, Skennerton CT, Hugenholtz P, Tyson GW (2015). CheckM: assessing the quality of microbial genomes recovered from isolates, single cells, and metagenomes. Genome Res..

[70] Chaumeil PA, Mussig AJ, Hugenholtz P, Parks DH. GTDB-Tk: a toolkit to classify genomes with the Genome Taxonomy Database. Bioinformatics. 2019;btz848. 10.1093/bioinformatics/btz848. 10.1093/bioinformatics/btz848 PMC7703759 31730192

[71] Quast C, Pruesse E, Yilmaz P, Gerken J, Schweer T, Yarza P (2013). The SILVA ribosomal RNA gene database project: improved data processing and web-based tools. Nucleic Acids Res..

[72] Hyatt D, Chen GL, Locascio PF, Land ML, Larimer FW, Hauser LJ (2010). Prodigal: prokaryotic gene recognition and translation initiation site identification. BMC Bioinformatics..

[73] Consortium U (2018). UniProt: a worldwide hub of protein knowledge. Nucleic Acids Res..

[74] Robinson MD, Oshlack A (2010). A scaling normalization method for differential expression analysis of RNA-seq data. Genome Biol..

[75] Hug LA, Castelle CJ, Wrighton KC, Thomas BC, Sharon I, Frischkorn KR (2013). Community genomic analyses constrain the distribution of metabolic traits across the Chloroflexi phylum and indicate roles in sediment carbon cycling. Microbiome..

[76] Novák L, Zubáčová Z, Karnkowska A, Kolisko M, Hroudová M, Stairs CW (2016). Arginine deiminase pathway enzymes: evolutionary history in metamonads and other eukaryotes. BMC Evol Biol.

[77] Edgar RC (2004). MUSCLE: multiple sequence alignment with high accuracy and high throughput. Nucleic Acids Res..

[78] Capella-Gutierrez S, Silla-Martinez JM, Gabaldon T (2009). trimAl: a tool for automated alignment trimming in large-scale phylogenetic analyses. Bioinformatics.

[79] Stamatakis A (2014). RAxML version 8: a tool for phylogenetic analysis and post-analysis of large phylogenies. Bioinformatics..

[80] Miller MA, Pfeiffer W, Schwartz T (2010). Creating the CIPRES Science Gateway for inference of large phylogenetic trees. 2010 Gateway Computing Environments Workshop (GCE).

[81] Letunic I, Bork P (2016). Interactive tree of life (iTOL) v3: an online tool for the display and annotation of phylogenetic and other trees. Nucleic Acids Res..

[82] Contreras-Moreira B, Vinuesa P (2013). GET_HOMOLOGUES, a versatile software package for scalable and robust microbial pangenome analysis. Appl Environ Microbiol..

