# Supplementary material for: Genomic and transcriptomic evidence of light-sensing, porphyrin biosynthesis, Calvin-Benson-Bassham cycle, and urea production in Bathyarchaeota
Source: Microbiome. 2020 Mar 31;8:43. doi: 10.1186/s40168-020-00820-1 (PMC7110647; doi:10.1186/s40168-020-00820-1)
Supplement: Supplementary file 4 — Additional file 3: Table S3. Metagenomic and transcriptomic coverage of bathyarchaeotal genomic bins. [file 40168_2020_820_MOESM3_ESM.docx]

**Table S3 Sequencing details of all samples**

| Samples | Depth (cm) | Metagenome size (bp) | Assembly size (bp) | No. of scaffolds | Transcriptome size (bp) |
| --- | --- | --- | --- | --- | --- |
| SZ_1 | 0-2 | 105 629 829 300 | 4 316 933 501 | 4 079 229 | 12 359 953 800 |
| SZ_2 | 6-8 | 121 140 789 000 | 4 600 435 761 | 4 448 659 | 12 365 197 800 |
| SZ_3 | 12-14 | 90 649 512 000 | 3 071 252 754 | 3 403 341 | 11 700 158 700 |
| Maipo-7 | 0-2 | 126 931 188 000 | 4 719 669 908 | 5 384 940 | 9 625 388 538 |
| Maipo-8 | 10-15 | 91 087 276 800 | 4 485 094 932 | 4 173 187 | 9 508 824 875 |
| Maipo-9 | 20-25 | 88 638 755 400 | 3 300 669 271 | 3 693 665 | 9 439 541 547 |
| Maipo-10 | 0-5 | 98 149 455 300 | 3 207 146 457 | 3 732 640 | 10 747 985 269 |
| Maipo-11 | 13-16 | 85 982 810 700 | 2 934 660 731 | 3 034 880 | 12 976 530 743 |
