# Supplementary material for: Genomic and transcriptomic evidence of light-sensing, porphyrin biosynthesis, Calvin-Benson-Bassham cycle, and urea production in Bathyarchaeota
Source: Microbiome. 2020 Mar 31;8:43. doi: 10.1186/s40168-020-00820-1 (PMC7110647; doi:10.1186/s40168-020-00820-1)
Supplement: Supplementary file 5 — Additional file 4: Table S4. Annotated results of the genes mentioned in the current study. [file 40168_2020_820_MOESM4_ESM.docx]

**Table S4 Detail information of bathyarchaeotal genomic bins**

| Bin Id | Completeness | Contamination | Strain heterogeneity | Total Length (bp) | No. of scaffolds | No. of genes | Avg. GC% |
| --- | --- | --- | --- | --- | --- | --- | --- |
| MF-10.3 | 95.33 | 3.27 | 0 | 1 591 409 | 183 | 1 669 | 43.23 |
| MF-10.5.5.11.1.24 | 91.98 | 5.19 | 0 | 1 820 428 | 302 | 2 036 | 41.80 |
| MF-9.11 | 73.06 | 2.34 | 25 | 637 791 | 140 | 729 | 46.16 |
| MF-6.8.11.21 | 58.03 | 1.55 | 0 | 1 152 944 | 416 | 1 487 | 58.90 |
| MF-5.3.1.4 | 73.23 | 0.97 | 0 | 1 214 426 | 348 | 1 459 | 42.94 |
| MF-4.7.5.8 | 83.64 | 1.01 | 0 | 1 015 914 | 376 | 1 258 | 34.68 |
| MF-3.4 | 60.77 | 2.58 | 0 | 609 036 | 178 | 727 | 41.58 |
| MF-4.4.10.23.1.6 | 77.88 | 1.62 | 60 | 1 944 508 | 613 | 2 415 | 50.41 |
| MF-4.2.1.10.12.7 | 85.83 | 7.32 | 0 | 1 702 528 | 400 | 1 918 | 41.77 |
