# Supplementary figures and images for: Genomic and transcriptomic evidence of light-sensing, porphyrin biosynthesis, Calvin-Benson-Bassham cycle, and urea production in Bathyarchaeota
Source: Microbiome. 2020 Mar 31;8:43. doi: 10.1186/s40168-020-00820-1 (PMC7110647; doi:10.1186/s40168-020-00820-1)

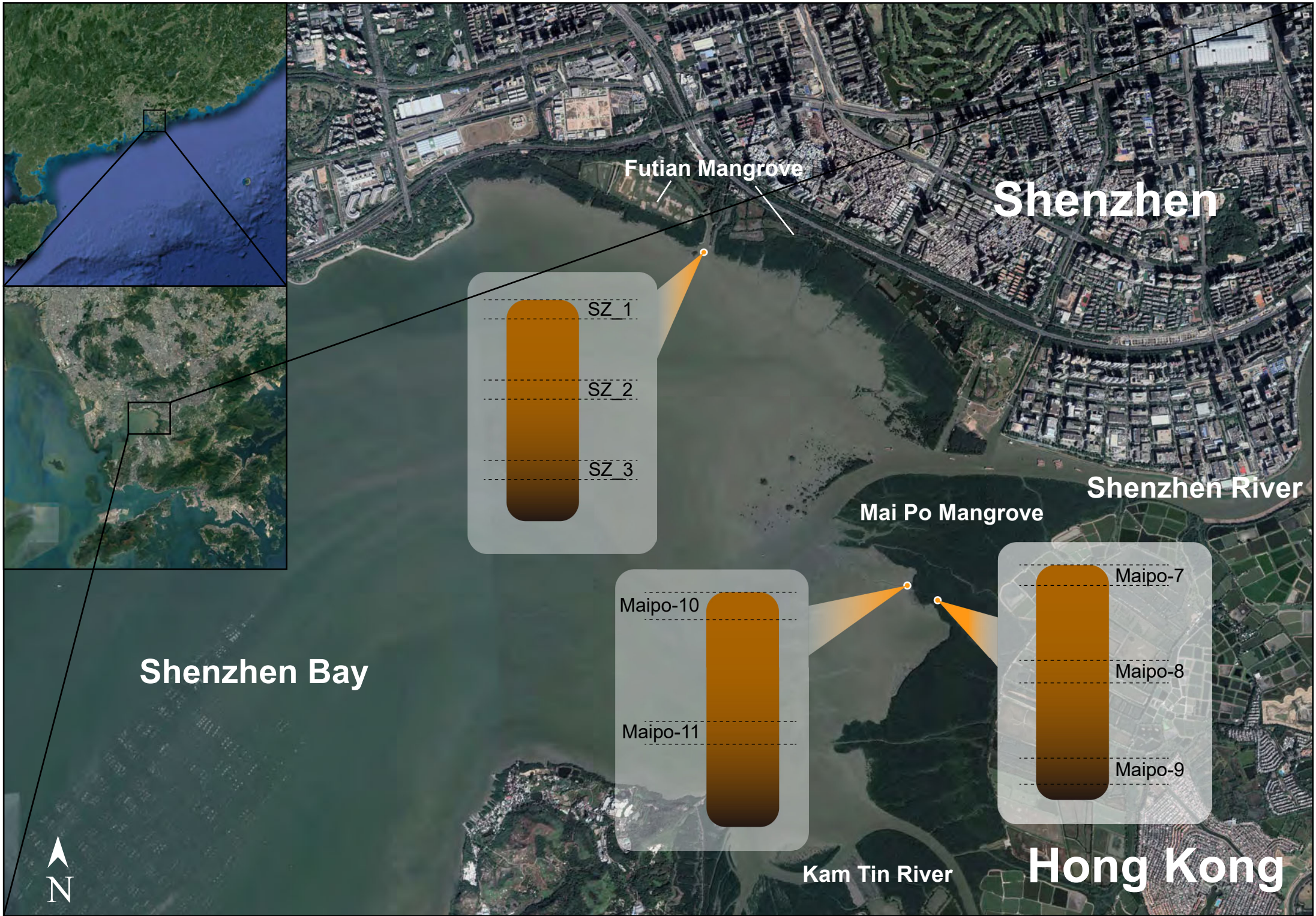

Supplement: Supplementary file 8 — Additional file 7: Figure S1. The location of the sample sites and the depths of the samples. [file 40168_2020_820_MOESM7_ESM.pdf]

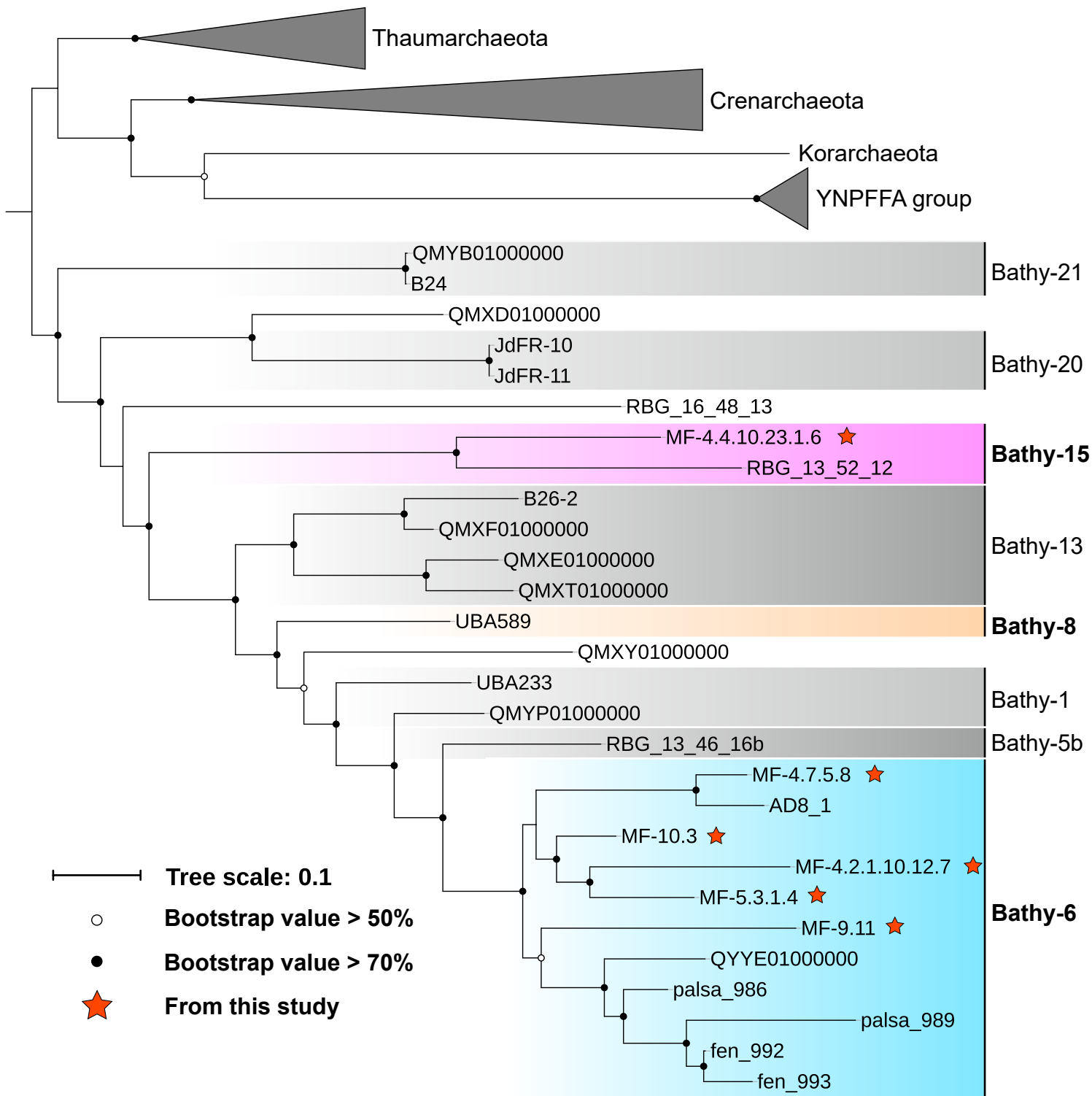

Supplement: Supplementary file 9 — Additional file 8: Figure S2. Subgroup assignment and phylogenetic tree of bathyarchaeotal genomes containing all 16 ribosomal proteins. [file 40168_2020_820_MOESM8_ESM.pdf]

# a. Metagenomic coverage

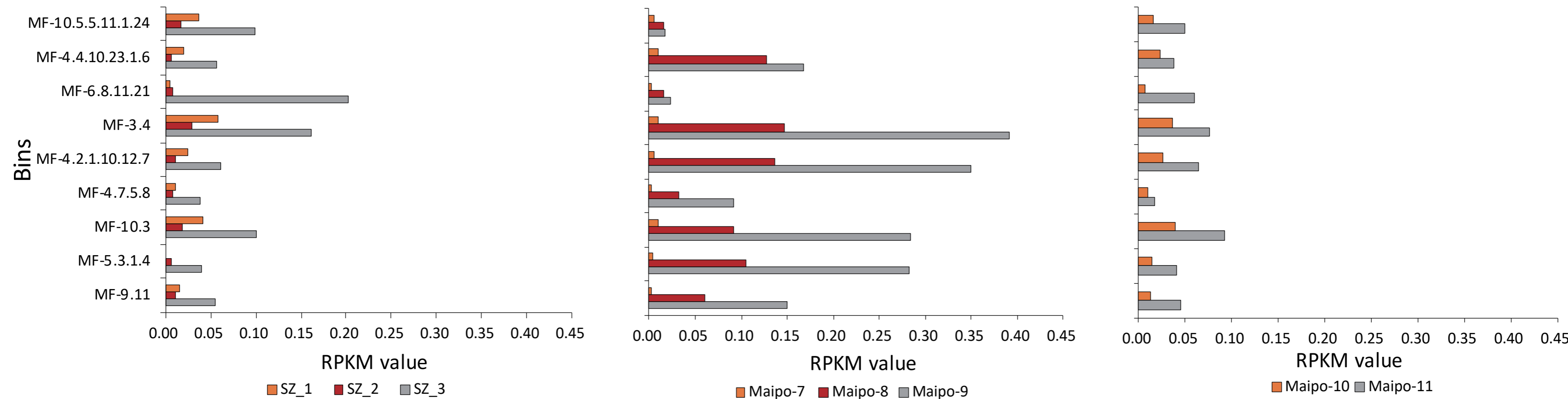

# b. Transcriptomic coverage

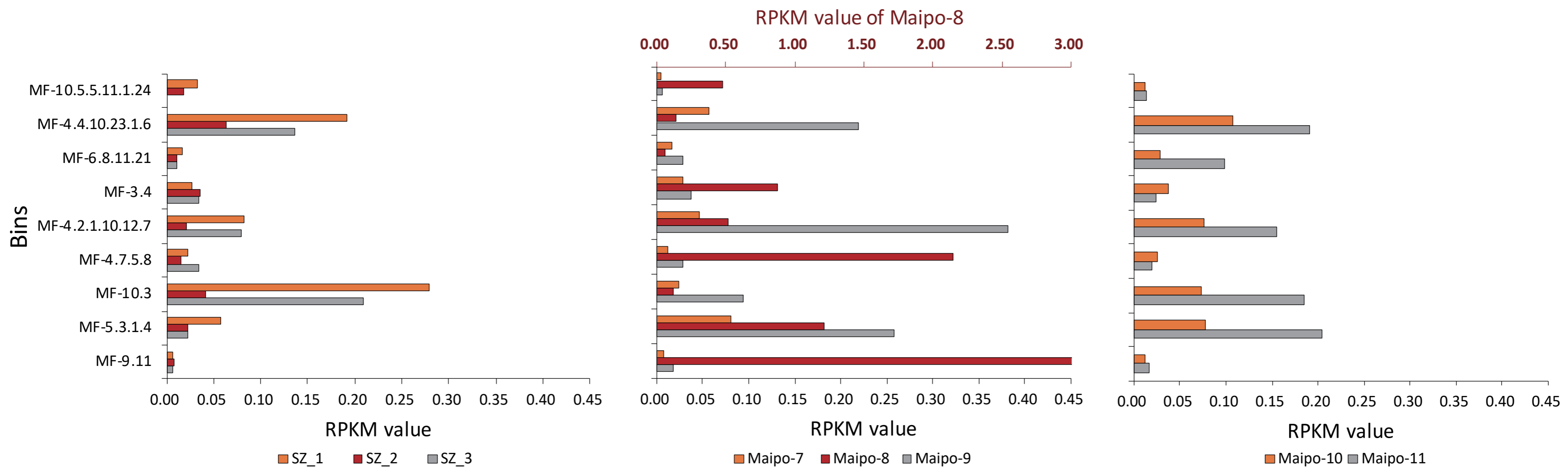

Supplement: Supplementary file 10 — Additional file 9: Figure S3. The metagenomic and transcriptomic coverages of nine bathyarchaeotal genomes in this study. [file 40168_2020_820_MOESM9_ESM.pdf]

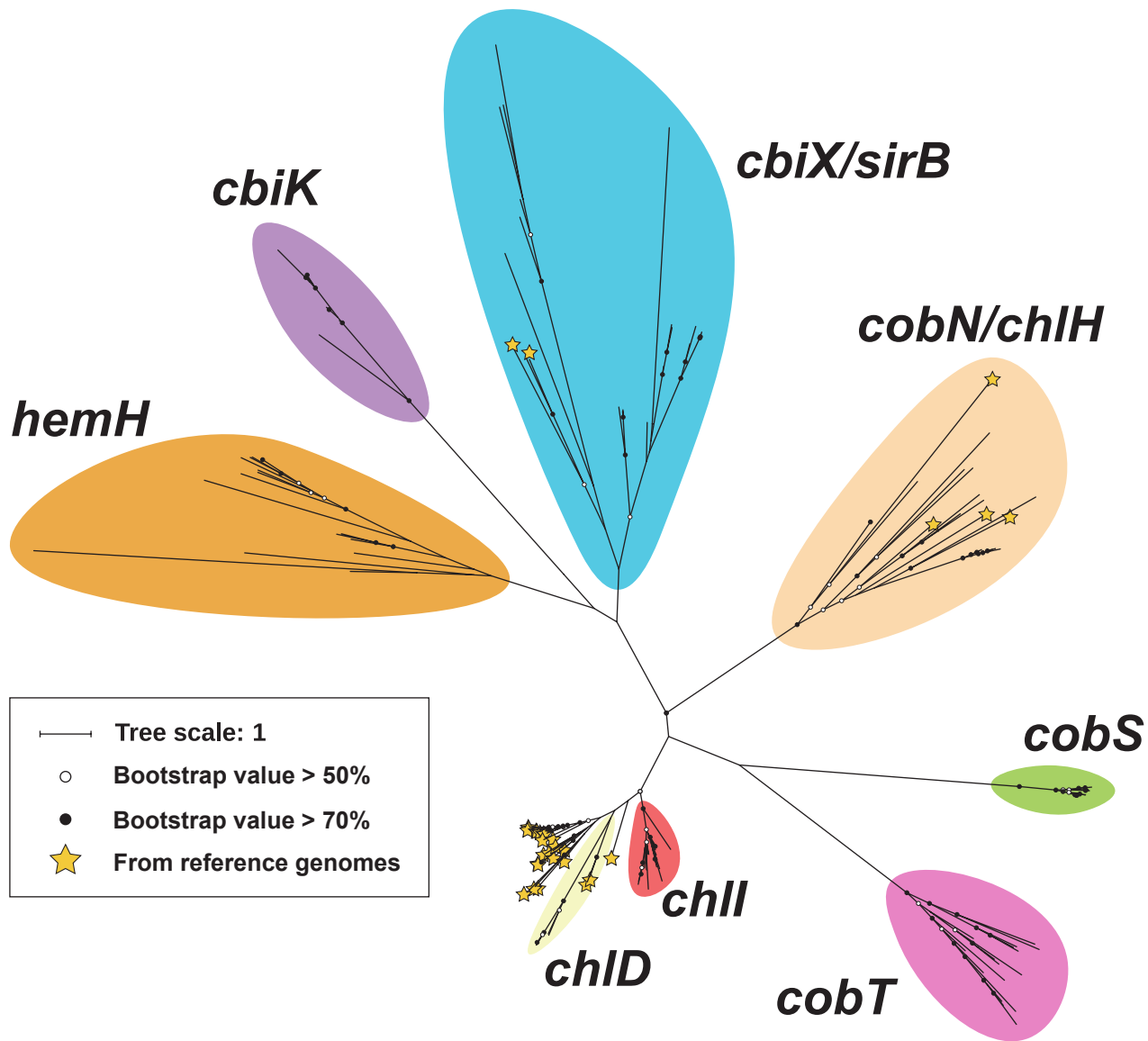

Supplement: Supplementary file 11 — Additional file 10: Figure S4. Maximum Likelihood tree of chelatase sequences. The scale bar indicates the average number of amino acid substitutions per site. The anchor sequences and methods are in Materials and methods. [file 40168_2020_820_MOESM10_ESM.pdf]

# Cu-Zn SOD Bacteria/Eukaryota

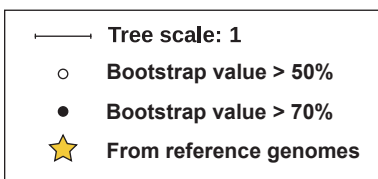

# Mn/Fe SOD Archaea

# Mn/Fe SOD Bacteria/Eukaryota

# Mn SOD Eukaryota

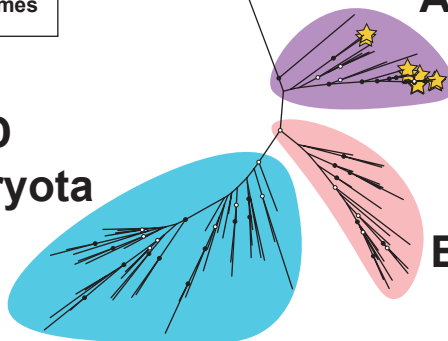

Supplement: Supplementary file 14 — Additional file 13: Figure S7. Maximum Likelihood tree of SOD sequences. The scale bar indicates the average number of amino acid substitutions per site. The anchor sequences and methods are in Materials and methods. [file 40168_2020_820_MOESM13_ESM.pdf]
