# Supplementary material for: Genomic and transcriptomic evidence of light-sensing, porphyrin biosynthesis, Calvin-Benson-Bassham cycle, and urea production in Bathyarchaeota
Source: Microbiome. 2020 Mar 31;8:43. doi: 10.1186/s40168-020-00820-1 (PMC7110647; doi:10.1186/s40168-020-00820-1)
Supplement: Supplementary file 12 — Additional file 11: Figure S5. Maximum Likelihood tree of PRK sequences. The scale bar indicates the average number of amino acid substitutions per site. The anchor sequences and methods are in Materials and methods. [file 40168_2020_820_MOESM11_ESM.pdf]

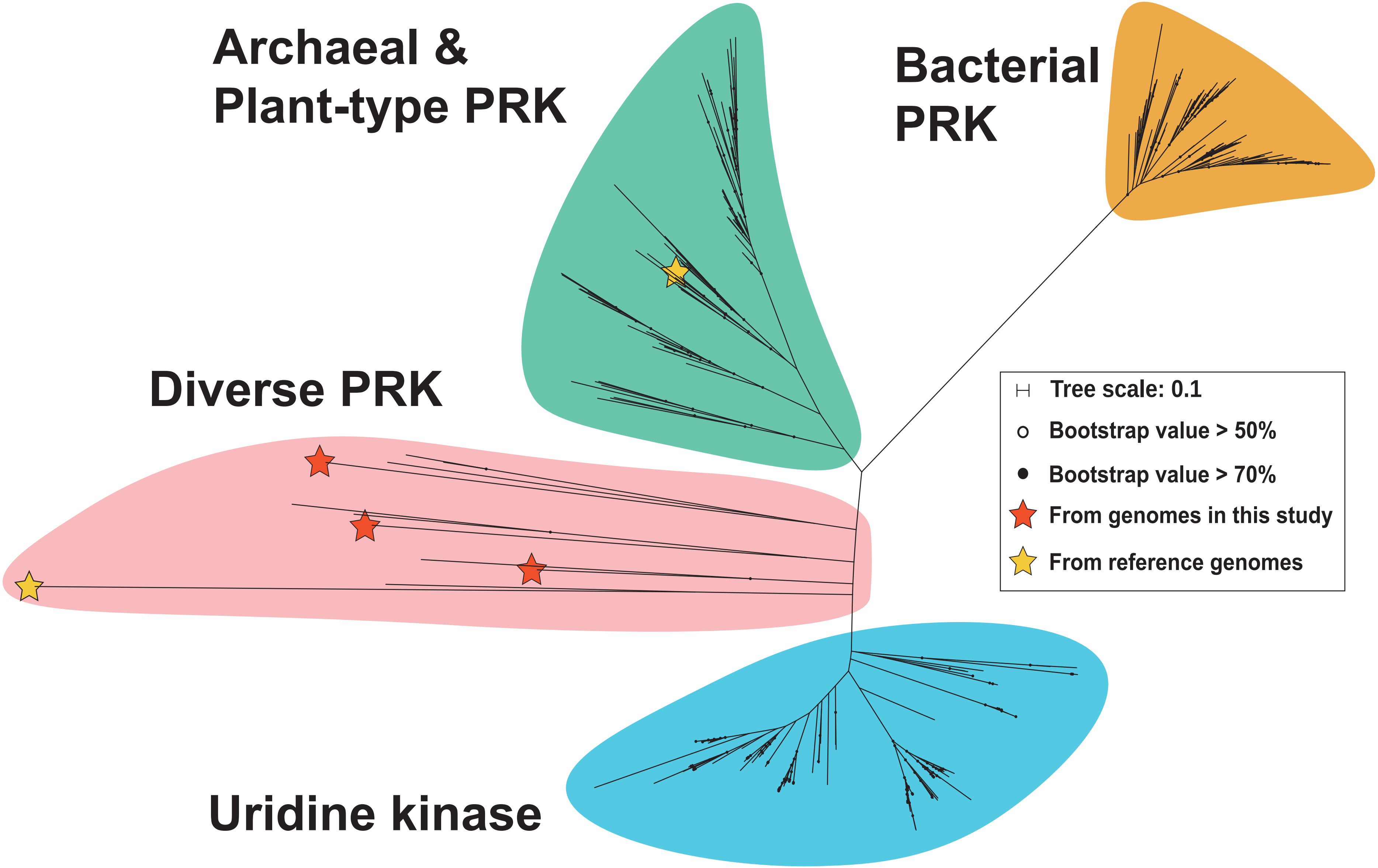
Archaeal &
Plant-type PRK
Bacterial
PRK
Diverse PRK
Uridine kinase
Tree scale: 0.1
Bootstrap value > 50%
Bootstrap value > 70%
From genomes in this study
From reference genomes
