# Supplementary material for: Genomic and transcriptomic evidence of light-sensing, porphyrin biosynthesis, Calvin-Benson-Bassham cycle, and urea production in Bathyarchaeota
Source: Microbiome. 2020 Mar 31;8:43. doi: 10.1186/s40168-020-00820-1 (PMC7110647; doi:10.1186/s40168-020-00820-1)
Supplement: Supplementary file 13 — Additional file 12: Figure S6. Maximum Likelihood tree of arginase and agmatinase sequences. The scale bar indicates the average number of amino acid substitutions per site. The anchor sequences and methods are in Materials and methods. [file 40168_2020_820_MOESM12_ESM.pdf]

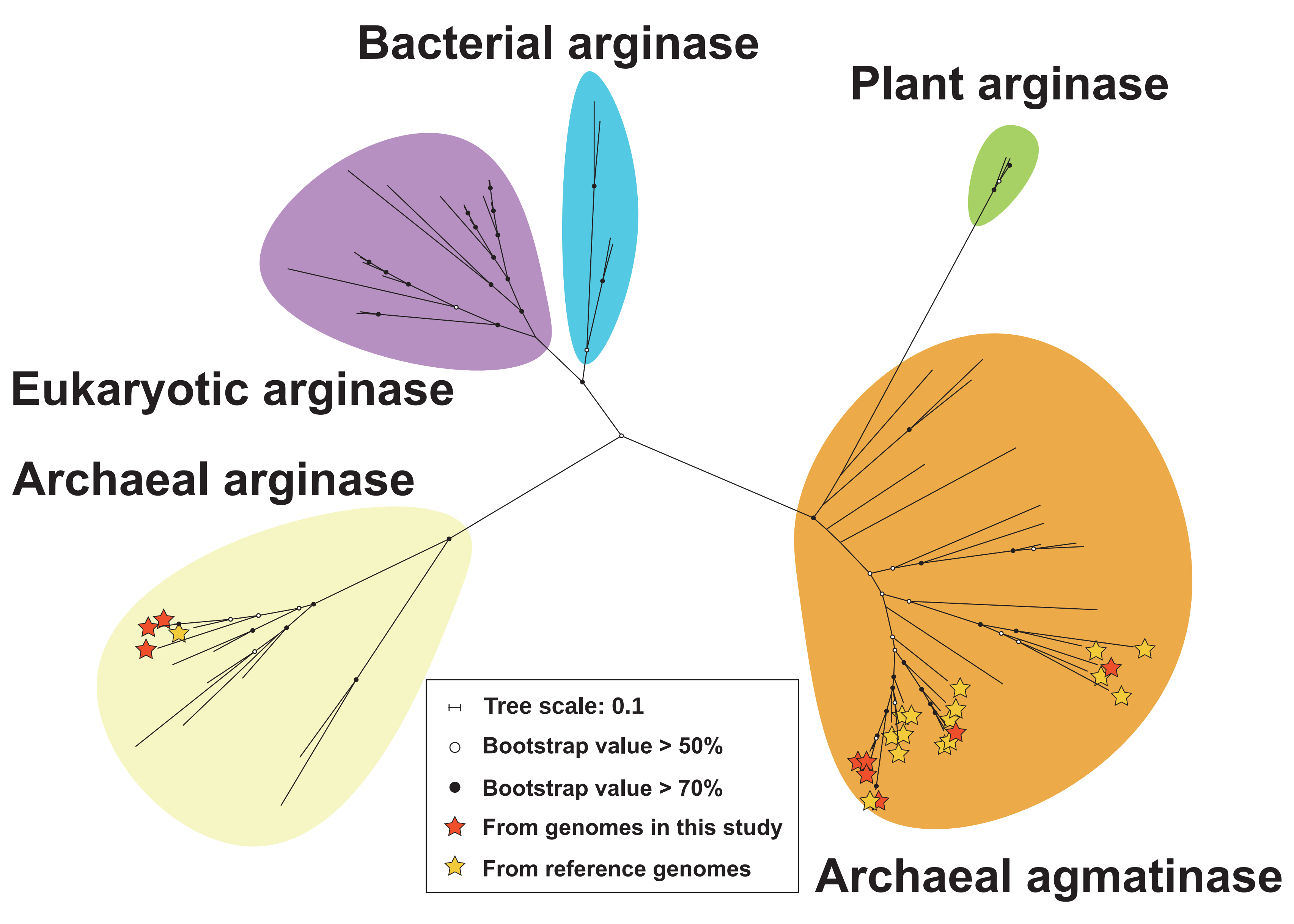

Bacterial arginase
Plant arginase
Eukaryotic arginase
Archaeal arginase
Archaeal agmatinase
Tree scale: 0.1
Bootstrap value > 50%
Bootstrap value > 70%
From genomes in this study
From reference genomes
