# Supplementary material for: Genomic and transcriptomic evidence of light-sensing, porphyrin biosynthesis, Calvin-Benson-Bassham cycle, and urea production in Bathyarchaeota
Source: Microbiome. 2020 Mar 31;8:43. doi: 10.1186/s40168-020-00820-1 (PMC7110647; doi:10.1186/s40168-020-00820-1)
Supplement: Supplementary file 15 — Additional file 14: Supplementary data 1. The explanation for gene abbreviations using in Fig. 2 and 4, Table S4, and S6. [file 40168_2020_820_MOESM14_ESM.docx]

Explanation for gene abbreviations in Figure 2, 4, S1, and S2

*fmd*/*fwd* formylmethanofuran dehydrogenase

*ftr* formylmethanofuran-tetrahydromethanopterin N-formyltransferase

*mch* methenyltetrahydromethanopterin cyclohydrolase

*mtd* methylenetetrahydromethanopterin dehydrogenase

*mer* 5,10-methylenetetrahydromethanopterin reductase

*mtdB* methylene-tetrahydromethanopterin dehydrogenase

*fdo*/*fdh*/*fdw* formate dehydrogenase

*fhs* formate--tetrahydrofolate ligase

*fold* methylenetetrahydrofolate dehydrogenase (NADP+)

*metF* methylenetetrahydrofolate reductase (NADPH)

*cdhC*/*acsC*/*cdhD* acetyl-CoA decarbonylase/synthase, CODH/ACS complex subunit

*cdhA*/*cdhB* acetyl-CoA decarbonylase/synthase, CODH/ACS complex subunit

*mttB*/*mtbB* trimethylamine---corrinoid protein Co-methyltransferase

*mtmB* methylamine---corrinoid protein Co-methyltransferase

*mtr* tetrahydromethanopterin S-methyltransferase

*mgs* methylamine---glutamate N-methyltransferase

*mgd* methylglutamate dehydrogenase

*fae*/*fae*−*hps* 5,6,7,8-tetrahydromethanopterin hydro-lyase

*prk* phosphoribulokinase

*rbcL* ribulose-bisphosphate carboxylase (RuBisCO)

*pgk* phosphoglycerate kinase

*gapA* glyceraldehyde 3-phosphate dehydrogenase

*tpiA* triosephosphate isomerase

*fba* fructose-bisphosphate aldolase

*fbp* fructose-1,6-bisphosphatase

*tkt* transketolase

*rpe* ribulose-phosphate 3-epimerase

*amt* ammonium transporter

*nifH* nitrogenase iron protein

*narH* nitrate reductase / nitrite oxidoreductase

*nir* nitrite reductase (NADH)

*hcp* hydroxylamine reductase

*carAB* carbamoyl-phosphate synthase

*argF* ornithine carbamoyltransferase

*argG* argininosuccinate synthase

*argH* argininosuccinate lyase

*rocF* arginase

*speB* agmatinase

*sat*/*cysDN* sulfate adenylyltransferase

*cysC* adenylylsulfate kinase

*cysH* phosphoadenosine phosphosulfate reductase

*cysI* sulfite reductase (NADPH) hemoprotein

*asr* anaerobic sulfite reductase

*phs* thiosulfate reductase

*hydA* sulfhydrogenase

*poxL* pyruvate oxidase

SOD superoxide dismutase

*gltX* glutamyl-tRNA synthetase

*hemA* glutamyl-tRNA reductase

*hemL* glutamate-1-semialdehyde 2,1-aminomutase

*hemB* porphobilinogen synthase

*hemC* hydroxymethylbilane synthase

*hemD* uroporphyrinogen-III synthase

*cysG*/*cobA* uroporphyrin-III C-methyltransferase

*cysG*/MET8 precorrin-2 dehydrogenase / sirohydrochlorin ferrochelatase

*cbiK*/*cbiX* sirohydrochlorin cobaltochelatase

*cbiL*/*cobI* precorrin-2/cobalt-factor-2 C20-methyltransferase

*cbiH*/*cobJ* cobalt-precorrin 5A hydrolase / precorrin-3B C17-methyltransferase

*cbiF*/*cobM* precorrin-4/cobalt-precorrin-4 C11-methyltransferase

*cbiG* cobalt-precorrin 5A hydrolase

*cbiD* cobalt-precorrin-5B (C1)-methyltransferase

*cbiJ*/*cobK* precorrin-6A/cobalt-precorrin-6A reductase

*cbiT* cobalt-precorrin-6B (C15)-methyltransferase

*cbiE* cobalt-precorrin-7 (C5)-methyltransferase

*cbiC*/*cobH* precorrin-8X/cobalt-precorrin-8 methylmutase

*cbiA*/*cobB* cobyrinic acid a,c-diamide synthase

*cobA*/*BtuR*/*pduO* cob(I)alamin adenosyltransferase

*cbiP*/*cobQ* adenosylcobyric acid synthase

*cbiB*/*cobC*/*cobD* adenosylcobinamide-phosphate synthase

*cobU*/*cobP*/*cobY* adenosylcobinamide-phosphate guanylyltransferase

*cobS*/*cobV* adenosylcobinamide-GDP ribazoletransferase

*bchM* magnesium-protoporphyrin O-methyltransferase

*bchE* anaerobic magnesium-protoporphyrin IX monomethyl ester cyclase

*dvr* divinyl chlorophyllide a 8-vinyl-reductase

*por* protochlorophyllide reductase

*bchG* chlorophyll/bacteriochlorophyll a synthase
